# Editorial: Recent advances and future directions in facial appearance research

**DOI:** 10.3389/fpsyg.2023.1154703

**Published:** 2023-02-22

**Authors:** Yoshiyuki Ueda, Koyo Nakamura, Chihiro Saegusa, Ayahito Ito

**Affiliations:** ^1^Institute for the Future of Human Society, Kyoto University, Kyoto, Japan; ^2^Faculty of Psychology, University of Vienna, Vienna, Austria; ^3^Japan Society for the Promotion of Science, Tokyo, Japan; ^4^Kao Corporation, Tokyo, Japan; ^5^Research Institute for Future Design, Kochi University of Technology, Kami, Japan; ^6^Department of Psychology, University of Southampton, Southampton, United Kingdom; ^7^Faculty of Health Sciences, Hokkaido University, Sapporo, Japan

**Keywords:** face, impression, judgment, social perception, representation, visualization, fMRI

This Research Topic aims to present readers with a group of papers that represent state-of-the-art facial appearance research. Faces are important in identifying individuals. However, they also significantly contribute to our estimation of the kind of a person someone is and a person's current emotions and moods. Owing to its social significance, facial appearance research has long been at the core of psychological research. The papers published in the Research Topic are very diverse and cover a variety of topics in this field. These studies include the internal representation of faces (Elson et al.; Minemoto and Ueda), personal characteristics inferred from faces (Albohn et al.; Ito et al.; Komori et al.; Mattavelli et al.; Sakuta; Ueda), visualization and face modeling (Albohn et al.; Komori et al.; Watson and Johnston), and impressions given by sanitary face masks (Dudarev et al.; Kamatani et al.). These methodologies include standard rating tasks (Dudarev et al.; Kamatani et al.; Mattavelli et al.; Sakuta), adaptation (Minemoto and Ueda), as well as visualization using principal component analysis (PCA; Watson and Johnston), StyleGAN (Albohn et al.), and Bayesian optimization (Komori et al.), and functional magnetic resonance imaging (fMRI; Elson et al.; Ito et al.; Ueda). Some studies have proposed a new methodology for facial appearance research (Albohn et al.; Watson and Johnston), created a new database (Mattavelli et al.), examined young children (Sakuta) and cultural differences (Dudarev et al.; Kamatani et al.; Sakuta), and reviewed previous studies (Minemoto and Ueda; Ueda).

The idea of “face space” (Valentine, 1991; Valentine et al., 2016) has been extensively used to understand the neural representation of faces. To understand the neural representation of faces distant from an average face, Elson et al. investigated the effects of caricaturing faces on neural responses using fMRI. Their findings indicate that the neural processing of caricatured faces recruits an object-selective cortex.

Adaptation and aftereffect methods attempt to examine face space in terms of behavioral indicators. Minemoto and Ueda reviewed recent research and showed whether the norm-based coding hypothesis, a theory for representing faces, can be applied to children and people with impaired face recognition. They also presented studies investigating the representation of ensembles (summary statistics) of faces and social signals via faces, suggesting a new direction for face adaptation paradigms.

Emerging evidence shows that the determination of trait impressions of faces can vary across individuals and cultures to a non-negligible extent (Hehman et al., 2017; Jones et al., 2021). Therefore, it is necessary to rethink the universality assumed in early theories. Sakuta attempted to test the universality of facial trait inferences by investigating young non-Western children and adults. The results showed that trustworthiness and competence inferred from facial appearance are culturally diverse, whereas dominance remains relatively shared across cultures.

Facial appearance influences various social relationships; early romantic feelings are no exception. Ueda marshaled previous literature on how human brains integrate perceived facial attractiveness into initial romantic attraction. He proposed that an important topic for future research is how human brains shape a persistent attraction to a particular person, which should be examined by integrating neuroimaging with ecologically valid tasks.

Regarding “cuteness,” another impression perceived from faces, Komori et al. aimed to identify the facial features related to this perception. They used Bayesian optimization, a global sequential optimization method for estimating unknown functions, to search for facial morphological features that enhance the perception of facial cuteness. The results showed that perceived cuteness was linked to a relatively lower position of facial components and narrower jawline, but not to forehead height.

Face evaluation and first impressions are influenced not only by invariant facial features but also by multiple other factors; however, the integrated modulation has not been systematically investigated. Mattavelli et al. created a new database, referred to as the Bi-AGI database, consisting of male and female faces of varying age, gaze direction, and illumination conditions, and found that illumination has a greater effect on face evaluation of younger faces. The new database is freely available.

A key to success in the scientific modeling of faces is to capture the facial features that appear in real faces. Albohn et al. introduced a novel paradigm for generating and visualizing photorealistic face images that correspond to an individual's mental representation. Previous research on faces broadly examined group-level consensus. However, individual differences are larger than the variability in stimulus levels and have limitations in that individual face representations are examined using noisy and blurred visualizations. Their proposed method can generate generalizable faces, thereby resulting in an understanding of how social judgments are formed.

Watson and Johnston proposed a PCA-based active-appearance model to capture spatiotemporal variations in dynamic facial expressions. Extending the existing face caricaturing techniques, this model allows the generation of dynamically caricatured and realistic faces.

To uncover the perceptual mechanisms of facial appearance, the characteristics of the face as well as those of the perceiver should be considered. Ito et al. investigated own-age bias in facial impression formation and noted a higher preference for young faces than for older faces, regardless of the age of participants. This suggests that preferential choice of face is less susceptible to own-age bias across the lifespan of individuals.

The perception of mask-wearing faces is gaining attention owing to their widespread use due to COVID-19. Recent studies have shown mixed results regarding whether wearing a sanitary mask enhances the perceived facial attractiveness. Kamatani et al. proposed a new hypothesis stating that the occluded area of the face is interpolated by a moderately attractive face shaped by each person's experience, and further examined it using the other-race effect.

Dudarev et al. aimed to elucidate the influence of wearing sanitary masks on the perceived attractiveness of the wearer. They showed that pro-mask participants rated individuals with masks as generally more attractive than individuals without masks, whereas anti-mask participants rated vice versa. These results suggest that perceived attractiveness is also affected by perceiver characteristics.

These studies will provide readers with useful contemporary psychological findings regarding facial appearance and the impressions derived from it. Furthermore, they provide not only new scientific insights but also novel and freely available methods and stimulus sets that will further elucidate the mechanisms of facial appearance processing. Facial appearance, for better or worse, governs human society. We hope this Research Topic contributes to clarifying how we make decisions and are biased regarding them.

## Author contributions

All authors listed have made a substantial, direct, and intellectual contribution to the work and approved it for publication.

## References

[B1] Hehman E. Sutherland C. A. Flake J. K. Slepian M. L. (2017). The unique contributions of perceiver and target characteristics in person perception. J. Pers. Soc. Psychol. 113, 513. 10.1037/pspa0000090 28481616

[B2] Jones B. C. DeBruine L. M. Flake J. K. Liuzza M. T. Antfolk J. Arinze N. C.. (2021). To which world regions does the valence–dominance model of social perception apply? Nat. Hum. Behav. 5, 159–169. 10.1038/s41562-020-01007-2 33398150

[B3] Valentine T. (1991). A unified account of the effects of distinctiveness, inversion, and race in face recognition. Q. J. Exp. Psychol. A. 43, 161–204. 10.1080/14640749108400966 1866456

[B4] Valentine T. Lewis M. B. Hills P. J. (2016). Face-space: a unifying concept in face recognition research. Q. J. Exp. Psychol. 69, 1996–2019. 10.1080/17470218.2014.990392 25427883

